# Chemical Reaction of Soybean Flavonoids with DNA: A Computational Study Using the Implicit Solvent Model

**DOI:** 10.3390/ijms13021269

**Published:** 2012-01-25

**Authors:** Hassan H. Abdallah, Janez Mavri, Matej Repič, Vannajan Sanghiran Lee, Habibah A. Wahab

**Affiliations:** 1 School of Chemical Sciences, University Sains Malaysia, Penang 11800, Malaysia; E-Mail: hwchems@yahoo.com; 2 National Institute of Chemistry, Hajdrihova 19, SI-1001 Ljubljana, P. O. Box 660, Slovenia; E-Mails: janez.mavri@ki.si (J.M.); matej.repic@ki.si (M.R.); 3 EN-FIST Centre of Excellence, Dunajska 156, SI-1000 Ljubljana, Slovenia; 4 Department of Chemistry, Faculty of Science, University of Malaya, Kuala Lumpur 50603, Malaysia; 5 School of Pharmaceutical Sciences, University Sains Malaysia, Penang 11800, Malaysia

**Keywords:** flavonoids, DNA, chemical reaction, carcinogenesis, DFT calculation

## Abstract

Genistein, daidzein, glycitein and quercetin are flavonoids present in soybean and other vegetables in high amounts. These flavonoids can be metabolically converted to more active forms, which may react with guanine in the DNA to form complexes and can lead to DNA depurination. We assumed two ultimate carcinogen forms of each of these flavonoids, diol epoxide form and diketone form. Density functional theory (DFT) and Hartree-Fock (HF) methods were used to study the reaction thermodynamics between active forms of flavonoids and DNA guanine. Solvent reaction field method of Tomasi and co-workers and the Langevin dipoles method of Florian and Warshel were used to calculate the hydration free energies. Activation free energy for each reaction was estimated using the linear free energy relation. Our calculations show that diol epoxide forms of flavonoids are more reactive than the corresponding diketone forms and are hence more likely flavonoid ultimate carcinogens. Genistein, daidzein and glycitein show comparable reactivity while quercetin is less reactive toward DNA.

## 1. Introduction

Carcinogenesis is a complex pathological process, where normal cells become neoplastic. It is mainly the process associated with chemical modification of DNA. Chemical modification of DNA could be caused by viruses, photochemical reactions or reactive substances, called carcinogens [1–4]. Carcinogens can either be of endogenous origin [5,6] (*i.e.*, hormones or their metabolites) or of exogenous origin [7] (*i.e.*, those chemicals that originate from the environment). The first step for chemically induced carcinogenicity is the alteration of the cellular DNA by the reactive form of carcinogen. Failure to repair DNA adducts can lead to depurination, as in the case of polyaromatic hydrocarbons [8], or to errors in DNA replication. Both introduce a mutation in DNA which can cause translocation and amplification of specific genes (proto-oncogenes), which translate into transformation from normal to altered cell. Indeed, multiple cumulative mutational events are invariably required for the progression from normal to fully malignant phenotype [9]. The altered cell may remain dormant or under specific circumstances may proliferate into paraneoplastic and ultimately progress to neoplastic cell. It is clear that repair mechanisms and the immune system play very important roles in carcinogenesis. The repair mechanisms in cells are very proficient, but there is evidence that they can be overwhelmed and consequently fail as well. For a recent review see [10].

Genistein, daidzein, glycitein and quercetin are flavonoids present in soybean [11] and other vegetables in high amounts, while quercetin is also sold as a dietary supplement. The majority of publications have focused on the inhibition effect of flavonoids on cell proliferation, although, experiments on the oral squamous cell line SCC-25 have shown a biphasic effect of quercetin on cell proliferation [12]. It was found that quercetin stimulated cell proliferation at concentrations up to 10 μM, whereas at higher concentrations, cell proliferation was inhibited and it is unclear under what conditions either of the effects prevail. Dihal and his group, has shown the ability of quercetin to inhibit the differentiation of Caco-2 cells in a behavior opposite to the anti-carcinogenic adducts with cellular protein and DNA in which, under certain conditions, cancer incidences are increased in experimental animals [13–19]. Some of these flavonoids exhibit estrogenic activity [20–22], but they are usually used for their powerful antioxidant [23] and chemopreventive properties [24–26]; their bioactivated forms are to a certain extent capable of reacting with DNA. Similar to polyaromatic hydrocarbons (PAHs), which are known carcinogens [27,28], flavonoids are not carcinogenic *per se*, but can become carcinogenic after bioactivation. The unmetabolized form is called procarcinogen, while the bioactivated metabolized form is called the ultimate carcinogen. PAHs [29,30], estrogens [31] and flavonoids are all metabolized by the family of cytochromes P450 to a wide variety of primary metabolites, including epoxides, dihydrodiols, quinones, and phenols [32–34], many of which can be reoxidized and recycled through the same metabolic pathways [29,30,35,36] giving rise to metabolites that are able to chemically react with DNA [27,30,37]. Studies on PAHs and estrogens have led us to conclude that the ultimate carcinogens of studied polyphenols can be either of diol epoxide or quinone type. This assumption is strengthened by studies that showed that the formation of quercetin’s quinone type metabolites is fast. The oxidations are performed by peroxidases (one electron oxidation) to form quercetin semiquinone radical and by tyrosinases (two electron oxidation) to form quercetin o-quinone [38,39].

The reaction responsible for the initial phase of carcinogenesis is alkylation of DNA by an ultimate carcinogen, typically at position N7 of guanine, although other alkylation sites are also possible [5]. It is well established, that guanine at position N7 is the most nucleophilic site of DNA and hence most likely to react [40]. Moreover, it was proven experimentally that large majority of the DNA chemical damages are associated with this site. Currently, quantum chemical calculations cannot treat the entire solvated DNA plus the ultimate carcinogen molecule into electronic details. Therefore we truncated the system to methylated guanine and the ultimate carcinogen, while the rest of the system was treated on the level of mean field theory on the Langevin dipoles and solvent reaction field levels. The approach proved to be successful and became a standard way of computational treatment for a large number of systems of biological interest. This approach represents an acceptable trade-off between physical relevance of the model and computational feasibility.

One strategy of preventing chemical damages of DNA is introduction of substances that react with the ultimate carcinogen faster than the ultimate carcinogen reacts with DNA [41]. Ascorbic acid and glutathione are well-known endogenous scavengers of ultimate carcinogens. The increased incidence of prostate cancer induced by complexation of glutathione by heavy metals is well documented in clinical practice [9]. Ellagic acid and polyphenols, such as flavonoids considered in this study, are also typically very efficient scavengers, but their bioactivated diol epoxide or quinone forms are nevertheless able to react with DNA. Thus it is not surprising that there have been studies concerning the safety of quercetin [42–45]. While many long-term studies showed that this compound is not carcinogenic [46,47] there are also contradicting results [48,49]. Moreover, it has been shown experimentally that genistein can mediate DNA strand breaks by H_2_O_2_/Cu(II) thus exerting influence on oxidative DNA damage [50]. It is worth to give comment on the accessibility of the DNA to the ultimate carcinogens. The ultimate carcinogens have no problems to reach densely packed genetic material. Biological macromolecules are soft and flexible and transport of the substrate to the reactive site is never the rate-limiting step.

The aim of this study was to critically examine the reactivity toward N7 position of guanine of four flavonoids and, for each of them, the both reactive forms were considered. Calculations were performed on Hartree-Fock and DFT level in conjunction with 6-31G(d) basis set. This double zeta basis set augmented with polarization functions on heavy atoms, is flexible enough to reliably describe the thermochemistry of the studied reactions. We considered the density functional B3LYP, where electron exchange is described by the method of Becke [51–53] and electron correlation by the methodology of Lee, Yang and Parr [54]. Structures were first optimized at the PM3 semiempirical MO, followed by HF and B3LYP calculations. Geometry optimizations were performed in the gas phase. For the reactive step we considered linear free energy relationship since the rate-limiting step involves proton transfer via solvent and locating the corresponding transition state is unfeasible. The effects of solvation were included by using solvent reaction field method of Tomasi and coworkers [55] and Langevin dipoles method of Florian and Warshel [56,57].

## 2. Results and Discussion

In this article, we considered two mechanisms for the DNA-adduct formation reaction according to the active form of the flavonoids. Since the selected flavonoids are structurally closely related, the linear free energy relation method was selected for our study. Moreover, the studied reactions are electrophilic substitutions with a complex mechanism involving proton transfer via several solvent molecules. Hence, locating the transition state and calculating the activation free energy for such a complex reaction is not practical. The application of linear free energy relationships to estimate the free energy of activation is well established and is described in reference [58]. In a series of chemical reactions involving similar reactants, which follow the same mechanism, the reaction with the most favorable reaction free energy will have the lowest free energy of activation. Detailed information on the two different proposed mechanisms and the type of adducts formed with guanine, as a model for DNA, using HF and DFT calculations, the chemical reaction will be discussed below.

### 2.1. Diol Epoxide Mechanism

The covalent binding of reactive intermediates to cellular DNA leading to adduct formation is considered to be a critical event in the initiation of carcinogenesis. Flavonoids may interfere with this process, by blocking the formation of reactive intermediates which can potentially prevent the initiation of carcinogenesis inhibiting the metabolic activation of the carcinogen to its reactive intermediates [59]. The effects of flavonoids on procarcinogen-activating enzymes, notably the cytochrome P450 CYP1 family, have been the focus of attention in cancer prevention during the last decade. For flavonoids to bind to DNA, these chemicals need bioactivation to a more reactive diol epoxide form that later attacks the N7 site of the DNA guanine to form DNA-adduct by enzymes such as cytochrome P450 (CYP) and epoxide hydrolase (EH) [60–62].

According to the diol epoxide mechanism the reaction proceeds in the following possible pathway. The N7 site of the DNA guanine approaches the epoxy ring carbon atom. During the reaction the strained epoxy ring opens, and formation of the chemical bond between the epoxy ring carbon atom and guanine is the rate-limiting step, followed by a fast proton transfer involving solvent molecules.

**Scheme 1:**
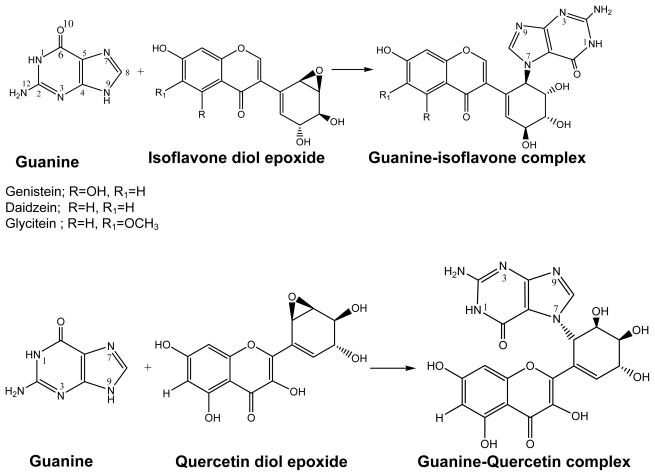
The diol epoxide mechanism for the DNA-adduct formation.

In this reaction, stereoisomeric epoxide reactant gives stereoisomeric product. These are soft degrees of freedom and the energy difference of several conformations is not large (<1 kcal/mol) and it is often difficult to decide simply by inspection which conformation will be adopted. Therefore, we have considered only one stereoisomer for comparison as shown in Scheme 1 for the entire calculation series. Besides, from previous linear free energy relationship study of benzo[*a*]pyrene diol epoxide stereoisomers [63], different conformations do not have much effect on reactivity.

The calculated reaction energy (Δ*E*), zero point energy correction (ZPE) and the reaction free energy for the flavonoids are listed in Table 1. The calculated reaction free energy for the chemical reaction calculated as the difference in energy between the reactants (flavonoids + guanine) and the product, which is the flavonoid-guanine complex (DNA adduct) from PCM was found to be lower than LD using both DFT and HF calculation for diol epoxide mechanism. The slightly lower values obtained from HF were found to be −27.98 (quercetin), −27.77 (genistein), −27.32 (daidzein), and −26.91 (glycitein) kcal/mol in similar trend agreed with DFT method among the calculated flavonoids. The negative sign indicates that the reaction is energetically favored. These values include the HF/6-31G(d) calculated classical barrier, 2.40–2.58 kcal/mol of calculated zero point energy correction and 1.48–2.23 kcal/mol of difference in free energy of hydration calculated using Tomasi’s approach. In comparison, the Langevin dipoles method for hydration free energy difference gave a value of 13.90–18.60 kcal/mol. It is clear that there is no significant difference between reaction free energies for the reactions of all active flavonoids with guanine. Neither *in vacuo* values of energies differ from each other nor the contributions from hydration free energies for both methods of calculation with the PCM solvent reaction field or the Langevin dipoles method.

**Figure 1:**
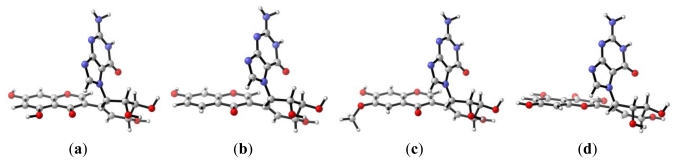
DFT optimized structures of the complex of the flavonoids diol epoxide-*N*7-guanine for (**a**) genistein-guanine, (**b**) daidzein-guanine, (**c**) glycitein-guanine, and (**d**) quercetin-guanine.

Experimental and theoretical results show that the polyaromatic hydrocarbons diol epoxide form is biologically active in the Fjord and Bay region and this activity is attributed to the high affinity for protonation and the high thermodynamic stability of the resulting cation [30,50]. According to our calculations, the diol epoxide forms of the flavonoids are highly active for attack on DNA, which may be attributed to the presence of the epoxide group. Figure 1 shows the geometry of the flavonoid-guanine complex according to the diol epoxide mechanism in which the guanine molecule has a nearly perpendicular position to the plane of the flavonoid molecule.

### 2.2. Diketone Mechanism

**Scheme 2:**
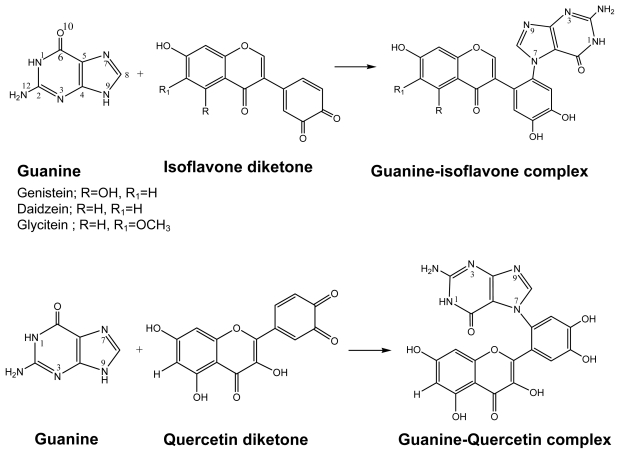
The diketone mechanism for the DNA-adducts formation.

The active form of the flavonoids in this reaction mechanism is in the diketone form and the reaction proceeds as in Scheme 2. The N7 site of the DNA guanine approaches the diene ring carbon atom, which is followed by the reduction of diketone to form the hydroxyl group. The calculated reaction free energy for the chemical reaction for DFT and HF are quite different. As a result in general, three isoflavones have a lower reaction free energy or the lower activation energy barrier in comparison to quercetin from diketone mechanism. However, with LD method using HF calculation, the diketone mechanism of all flavonoids is not favorable with positive reaction free energy. Also, quercetin appears with the most negative reaction free energy (−18.18 kcal/mol) on DFT with PCM solvation model. Calculations of hydration free energy on the HF/6-31g(d) level of theory exaggerates with the dipole moment and it corresponds to the polarized case as is the situation in aqueous solution. Langevine dipoles model was also parametrized for this level and hydration free energies are quite reliable. B3LYP functional systematically underestimates the reaction barrier due to absence of parametrization set for diketone species in the calculation as it is observed in PCM model for isoflavones with −11.44 to −11.79 kcal/mol (DFT) and −14.83 to −20.34 kcal/mol (HF). These values in B3LYP/6-31G(d) level also include the calculated classical barrier, 1.84–3.73 kcal/mol of B3LYP/6-31G(d) calculated zero point energy correction and 1.32–12.40 kcal/mol of difference in free energy of hydration calculated using the Langevin dipoles method as implemented in program ChemSol 2.1. In comparison, Tomasi’s approach for hydration free energy difference gave a value of 0.42–11.31 kcal/mol. It is also clear that there is no significant difference between reaction free energies for the reactions of all active flavonoids with guanine under this mechanism either *in vacuo* or with solvation contribution.

According to the values of the reaction energy and the reaction free energy diketone metabolites are less reactive than the diol epoxide. The diol epoxide mechanism shows no activity difference among flavonoids either in gas or solvent calculation. The calculated zero point contributions to reaction free energies for the three isoflavones are essentially identical, whereas a slightly higher value found for quercetin**,** as free energy of hydration was modeled on two levels of theory, PCM and LD. As parametrization of the Langevin dipole (LD) model is developed for quantum calculations of chemical processes in aqueous solution, the extension of the model to other polar solvents would require some simple reparameterization. The implementation and parametrization for aqueous solution was described with the training set that encompassed solvation free energies of 44 neutral and 39 ionic solutes of the C, O, N, P, S, F, and Cl atoms and was found to be comparable or slightly better than the PCM continuum model of Tomasi and co-workers to predict the acid dissociation constants (p*K*_a_) of the chemicals involved in proton transfer. Simplified explicit representation of solvent molecules of the LD model gives better insight into the molecular origin of different solvent effects than that obtained by continuum models. Solvent in LD model is approximated by polarizable discrete dipoles fixed on a cubic grid whereas the Polarizable Continuum Model calculates the molecular free energy in solution as the sum over the electrostatic, the dispersion-repulsion contributions to the free energy, and the cavitation using a cavity defined through interlocking *van der Waals* spheres centered at atomic positions. The three flavonoids (genistein, glycitein, and daidzein) according to the first mechanism show no substantial difference in hydration free energy. Interestingly, we noticed that quercetin according to both mechanisms on DFT with LD method needed more energy for hydration in comparison with genistein, glycitein, and daidzein. This behavior can be attributed to the extra hydroxyl group with higher polarization and electron correlation effect. The comparison of the values of the total energy in the gas phase by using HF and DFT theory and the values of the reaction free energy between the two mechanisms clearly showed that the first mechanism gives better results.

Comparing the two different calculation methods from Table 1, we achieved similar barrier heights in gas phase in diol epoxide mechanism, 29.66–31.95 kcal/mole for DFT and 31.16–32.79 kcal/mol for HF, however, application of HF significantly reduced the barrier (14.45–23.30 kcal/mol) in diketone mechanism. In previous work on alkylation of guanine by styrene-7,8-oxide, the combination of Hartree-Fock calculation using flexible basis sets and Langevin dipoles calculation of hydration free energies gives very reasonable agreement with the experimental free energy (26.52 kcal/mol) whereas B3LYP calculations predict a lower activation free energy [64]. We have the impression that the applied DFT method outperforms the HF calculations. Strong evidence indicates that for diketone mechanism, with hydration contributions included, the free energies are positive. The DFT calculated reaction free energies are lower for epoxide mechanism than for diketone mechanism for all the species. Therefore it is very likely, that the carcinogenic reaction proceeds via epoxide mechanism unless flavonoids activation by P450 is the rate-limiting step. Our calculations give strong evidence that genistein, daidzein and glycitein are basically equally reactive toward DNA, while quercetin has different reactivity, less reactive on DFT or more reactive on HF with LD solvation model. This is a possible reason why isoflavones may interfere with the promotion stage of carcinogenesis by blocking the formation of the metabolic activation of the carcinogen to its reactive intermediates. Thus, it remains a major challenge to fine tune the reactivity of quercetin analogs to widen the therapeutic window.

Finally, we can conclude that chemical reactions for four selected flavonoids either with diol epoxide or diketone for this, since free reaction energies are quite similar for all four flavonoid diol epoxides. One possible explanation is that different metabolic transformations at different rates may be the source of discrepancies in carcinogenicity. Other metabolic transformations at different rates could be found in particular reactions catalyzed by enzymes such as catechol-*O*-methyl transferase (COMT), glutathione-*S*-transferase, P450 (CYP families), aromatase and various peroxidases [63,65]. In addition, different reaction rates may occur in DNA depurination step, which would also result in different carcinogenicity profiles of studied compounds. Moreover, not only DNA, but proteins, as well as cellular lipids and some metallic ions (iron and copper) are targets for reactions with quinines [66,67]. It is therefore clear that reaction rates of metabolic transformations, adduct formation, depurination and reactivity with other cellular components all contribute to the overall carcinogenicity of studied compounds. The molecular modeling of chemical reactivity of each of these steps will play an important role in cancer research and will ultimately contribute to the prevention and improvement of cancer treatment.

## 3. Computational Detail

Initial structures of the flavonoids and the flavonoid-guanine adducts were built using Molden program. PM3 semiempirical MO method was used to refine the starting structures, which were submitted to the HF and DFT optimization with 6-31G(d) basis set. No constraints were applied. The double-zeta basis set augmented with polarization functions on heavy atoms is flexible enough to describe chemical processes and is also computationally tractable. To ensure that the optimized structure is the real minima rather than saddle points, vibrational analysis was performed in the harmonic approximation and the absence of imaginary frequencies proves that the stationary points are real minima rather that the saddle points. To consider the solvent effect on the reaction mechanism, the polarizable continuum model of Tomasi and coworkers as implemented in Gaussian03 was applied. Moreover, Langevin dipoles (LD) method in which the solute is described by a set of point charges on the grid of the Langevin dipoles, together with a proper parameterization, has received attention during the past years to model solvation effects. Langevin dipoles calculations were performed using CHEMSOL versions 2.1 package kindly provided by Jan Florian. The Merz-Kollman charges were calculated at B3LYP/6-31G(d) level using Tomasi’s PCM SCRF.

The reaction free energy was calculated as the free energy difference between products and the reactants corrected for zero point energy contribution calculated in the harmonic approximation and the hydration free energy.

$$\Delta G_{\text{react}}=\Delta E+\Delta \text{ZPE}+\Delta G_{\text{hydr}}$$

Langevin dipoles and Gaussian03 [68] calculations were performed on a dual-CPU PC/Linux cluster.

## 4. Conclusions

Reaction free energy and the free energy of hydration were calculated for four flavonoids present in soybean: genistein, daidzein, glycitein, and quercetin, for their reaction with guanine according to two kinds of mechanisms; diol epoxide and diketone mechanism. HF and DFT levels of theory in conjunction with 6-31G(d) basis set were employed for calculating the reaction energies *in vacuo.* LD and PCM solvation models were used for calculation of hydration free energies thus enabling the calculation of reaction free energies. This work sheds light on the mechanism of action of flavonoids. The results show that the diol epoxide mechanism describes the carcinogenic reaction better than the diketone mechanism. Our study shows that metabolites of soy food products are slightly reactive toward DNA but flavonoids are even more reactive toward the ultimate carcinogens of the epoxy type. In terms of cancer prevention, it is still beneficial to consume soy products. Quantum chemical calculations are valuable for the design of flavonoids derivatives with even lower activation energy and increased reactivity toward ultimate carcinogens as well as controlled reactivity toward DNA.

## Figures and Tables

**Figure 2:**
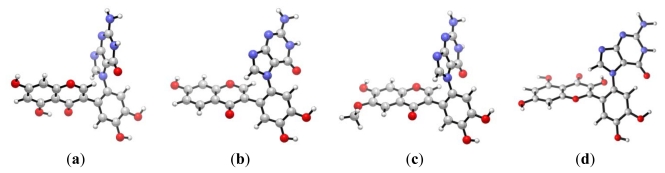
DFT optimized structures of the diketone flavonoid-*N*7-guanine complexes for (**a**) genistein-guanine, (**b**) daidzein-guanine, (**c**) glycitein-guanine, and (**d**) quercetin-guanine.

**Table 1:** Free energy components for reactions via diol epoxide and diketone mechanism with density functional theory (DFT) and Hartree-Fock (HF) calculation.

DFT Method (B3LYP/6-31G(d))						
	Δ*E*^a^	ΔZPE b	PCM method		LD method	
			Δ*G*_hydr_^c^	Δ*G*_react_^d^	Δ*G*_hydr_	Δ*G*_react_
**Diol epoxide mechanism**						
Genistein	−30.04	2.19	1.34	−26.51	6.50	−21.35
Daidzein	−29.74	2.21	1.20	−26.33	5.80	−21.73
Glycitein	−29.66	2.24	1.85	−25.57	5.90	−21.52
Quercetin	−31.95	2.55	1.52	−27.88	11.10	−18.30
**Diketone mechanism**						
Genistein	−26.83	3.73	11.31	−11.79	5.82	−17.28
Daidzein	−22.79	3.50	7.85	−11.44	3.28	−16.01
Glycitein	−22.94	3.50	7.65	−11.79	1.32	−18.12
Quercetin	−20.44	1.84	0.42	−18.18	12.40	−6.20
**HF Method (HF/6-31G(d))**						
	Δ*E*^a^	ΔZPE b	PCM method		LD method	
			Δ*G*_hydr_^c^	Δ*G*_react_^d^	Δ*G*_hydr_	Δ*G*_react_
**Diol epoxide mechanism**						
Genistein	−31.68	2.43	1.48	−27.77	18.60	−10.65
Daidzein	−31.26	2.40	1.54	−27.32	17.00	−11.86
Glycitein	−31.16	2.40	1.85	−26.91	15.10	−13.66
Quercetin	−32.79	2.58	2.23	−27.98	13.90	−16.31
**Diketone mechanism**						
Genistein	−23.30	1.57	1.39	−20.34	23.30	1.57
Daidzein	−17.58	1.59	0.93	−15.06	17.70	1.71
Glycitein	−17.49	1.60	1.06	−14.83	21.70	5.81
Quercetin	−14.45	1.52	1.06	−11.87	14.50	1.57

a Energy difference between the reactants (flavonoids + guanine) energy and the product which is the flavonoid-guanine complex (DNA adduct) in the gas phase based on B3LYP/6-31G(d);

b Zero point energy (ZPE) corrections;

c Δ*G*_hydr_, free energy of hydration differences;

d Δ*G*_react_, reaction free energy, Δ*G*_react_ = Δ*E* + ΔZPE + Δ*G*_hydr_. All energies are in kcal/mol.
